# *EXPANSIN15* is involved in flower and fruit development in Arabidopsis

**DOI:** 10.1007/s00497-023-00493-4

**Published:** 2024-01-29

**Authors:** Judith Jazmin Bernal-Gallardo, Karla L. González-Aguilera, Stefan de Folter

**Affiliations:** Unidad de Genómica Avanzada (UGA-Langebio), Centro de Investigación y de Estudios Avanzados del Instituto Politécnico Nacional (CINVESTAV-IPN), 36824 Irapuato, GTO. Mexico

**Keywords:** *Expansin15*, Flower, Gynoecium, Fruit, Transcription factors, Petal

## Abstract

**Key message:**

*EXPANSIN15* is involved in petal cell morphology and size, the fusion of the medial tissues in the gynoecium and expansion of fruit valve cells. It genetically interacts with *SPATULA* and *FRUITFULL*.

**Abstract:**

Cell expansion is fundamental for the formation of plant tissues and organs, contributing to their final shape and size during development. To better understand this process in flower and fruit development, we have studied the *EXPANSIN15* (*EXPA15)* gene, which showed expression in petals and in the gynoecium. By analyzing *expa15* mutant alleles, we found that EXPA15 is involved in petal shape and size determination, by affecting cell morphology and number. EXPA15 also has a function in fruit size, by affecting cell size and number. Furthermore, EXPA15 promotes fusion of the medial tissues in the gynoecium. In addition, we observed genetic interactions with the transcription factors SPATULA (SPT) and FRUITFULL (FUL) in gynoecium medial tissue fusion, style and stigma development and fruit development in Arabidopsis. These findings contribute to the importance of *EXPANSINS* in floral and fruit development in Arabidopsis.

**Supplementary Information:**

The online version contains supplementary material available at 10.1007/s00497-023-00493-4.

## Introduction

The size and shape of plant organs are regulated by proliferation and subsequent expansion, contributing to cell morphology in each organ (Ramachandran et al. 2000; Marshall et al. 2012; Guerriero et al. 2014; D’Ario et al. 2021). Cell wall expansion is a process mediated by EXPANSIN proteins, these enzymes are plant cell wall-loosening proteins that loosen cell walls by weakening the binding of polysaccharide polymers to each other, contributing to cell enlargement and thus to cell growth and shape (Sampedro and Cosgrove 2005).

According to expression patterns in Arabidopsis and other species, it is known that EXPANSIN proteins are expressed in floral tissues, such as sepals, petals, stamens and carpels, as well as in floral meristems (Brummell et al. 1999b; Armezzani et al. 2018; Liu et al. 2021). Expansins play an important role in the development of leaves, fruit, pollen tube and roots, as well as in defense against different stresses, in Arabidopsis and other species (Reinhardt et al. 1998; Brummell et al. 1999a; Chen and Bradford 2000; Cho and Cosgrove 2000; Wrobel and Yoder 2001; Pezzotti et al. 2002; Zenoni et al. 2004; Jones and McQueen-Mason 2004; Belfield et al. 2005; Balestrini et al. 2005; Giordano and Hirsch 2007; Tsuchiya et al. 2015; Muthusamy et al. 2020; Liu et al. 2021; Samalova et al. 2022, 2023). However, in Arabidopsis due to the redundancy of the EXPANSIN family, there are few examples of specific functions in Arabidopsis development (Sampedro and Cosgrove 2005; Marowa et al. 2016; Samalova et al. 2022).

On the other hand, specific transcription factor networks involved in Arabidopsis gynoecium and fruit development have been described (Chávez Montes et al. 2015; Herrera-Ubaldo and de Folter 2022; Herrera-Ubaldo et al. 2023). One of the transcription factors required for gynoecium development is SPATULA (SPT), which is known to be involved in the development of the medial tissues of the gynoecium, and the lack of its functions affects reproduction and consequently, fruit size (Alvarez and Smyth 1999; Heisler et al. 2001; Girin et al. 2011; Reymond et al. 2012; Reyes-Olalde et al. 2017a). Another transcription factor that affects fruit size is FRUITFULL (FUL) (Qing Gu et al. 1998; Ferrándiz et al. 2000). It has been reported that *SPT* through genetic interaction with *FUL*, participates also in fruit development (Groszmann et al. 2011), suggesting being involved in proliferation and expansion processes.

In this work, we focus on the enzyme encoding gene *EXPA15* in Arabidopsis flower and fruit development. In the flower, we found that *EXPA15* is involved in petal shape and size by affecting cell morphology, size and number. In addition, this enzyme is involved in fruit size by controlling expansion in fruit valve cells. Furthermore, we identified genetic interactions of *EXPA15* with the transcription factors *SPT* and *FUL*.

## Results

### Mutations in *EXPA15* affect petal cell morphology and number in Arabidopsis flowers

The enzyme encoding *EXPANSIN15* (*EXPA15*) gene has been described as an important developmental regulator in the meristem and is known to be expressed in the root (Armezzani et al. 2018; Samalova et al. 2023) but has not been described to play a role in floral development in Arabidopsis.

**Fig. 1:**
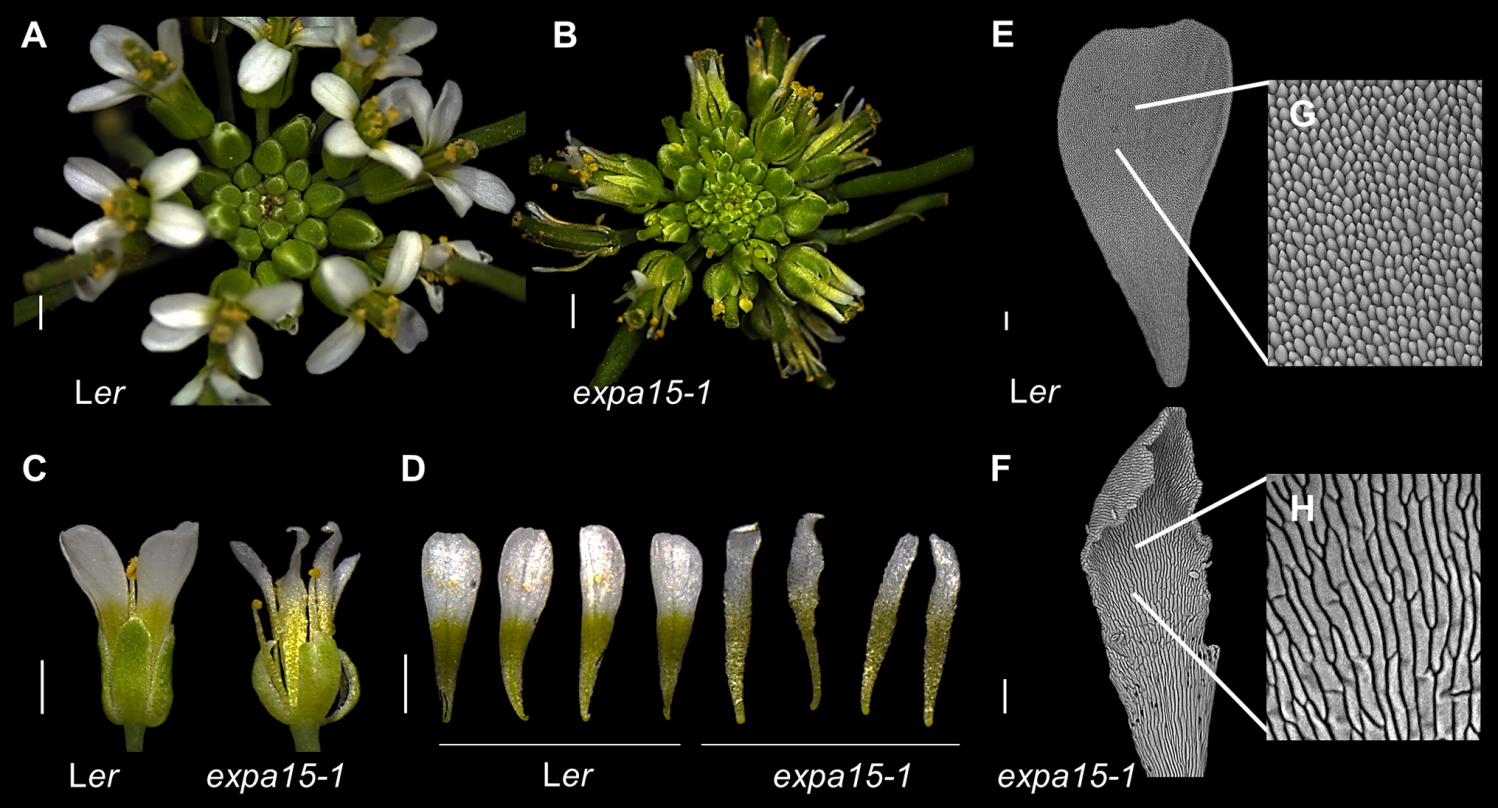
*EXPA15* affects petal cell morphology.**A**–**B** Inflorescence of L*er* and the *expa15-1* mutant. **C** Flower of wild-type L*er* and *expa15-1*. **D** Narrow petals in *expa15-1* compared to L*er*. **E**–**H**) Scanning microscopy images of L*er* and *expa15-1* in adaxial part of petals. **G** and **H** are magnifications of **E** and **F**. Scale bars: 1 mm in **A**–**D**; 200 µm in **E**, **F**

In this work, we analyzed the possible effects of the absence of *EXPA15* in flower and fruit development. We observed that in the inflorescences of *expa15-1*, there is a malformation in the flower (Fig. 1A–C), in part also observed in two additional homozygous insertional mutants in the Col-0 background, *expa15-2* and *expa15-3* (Figs. S1, S2, S3). In detail, the petals of the null mutant *expa15-1* mutant are narrower compared to the wild-type L*er* (WT) (Fig. 1D). At the cellular level, scanning micrographs show that petal cells in WT, which are characterized by their conical shape, lost this morphology in the *expa15-1* mutant, as the cells observed are flat and elongated (Fig. 1E–H). In addition, cell number is strongly reduced in the petal claw and blade (Fig. S4). Petal cell number is also reduced in the *expa15-2* and *expa15-3* mutants, though conical cell shape in petals is maintained (Figs. S2, S3, S4).

### *EXPA15* contributes to the development of the fruit by participating in cell expansion of the valves

**Fig. 2:**
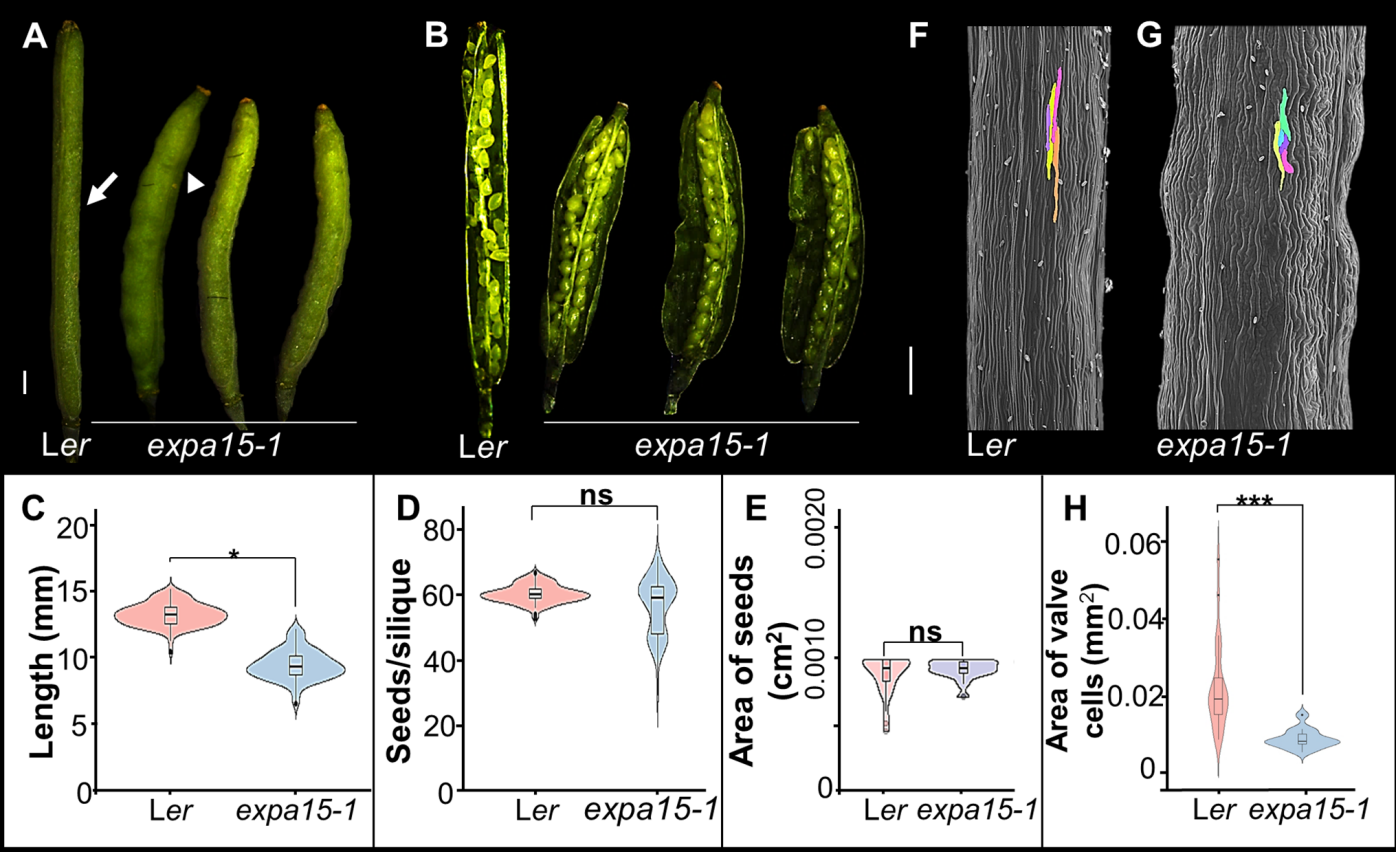
The mutation in the *EXPA*15 gene affects fruit length due to a decrease in the expansion of valve cells. **A**–**B** Images of fruits of L*er* and *expa15-1*. **C**–**D** Analysis on fruits and seeds of L*er* and *expa15-1*. **C** Fruit length. **D** Number of seeds per silique. **E** Area of seeds. **F**–**G** Scanning microscopy images of L*er* and *expa15-1* fruits. **H** Analysis of fruit valve cell area in L*er* and *expa15-1*. Statistical analyses were performed using a Wilcoxon test in **C**, **D**, **H** and Student´s *t*-test in **E**. *n* = 80 in **C**–**D**, *n* = 30 in **E**, *n* = 5 in **H**: *p < 0.01, ***p < 0.0001, ns: not significant, *p* > 0.05. Scale bars: 1 mm in **A**–**B**; 200 µm in **F**, **G**

Another phenotype observed in the *expa15* mutants is in fruit development. The *expa15-1* plants produce statistically significant shorter fruits (Fig. 2C), and moreover, the fruits have valves that are irregularly shaped, with bulging regions, something not observed in the WT (Fig. 2A, B). Furthermore, neither seed number nor seed area (Fig. 2D, E) are affected in *expa15-1* compared with WT. Probably, the bulging of the valves is caused by the seeds that are packed in a smaller volume, something similar has been observed in the mutant for the *FRUITFULL* (*FUL*) gene (Gu et al. 1998). To better understand the valve phenotype, we used scanning electron microscopy (SEM) and found that fruit valve cells are smaller in the *expa15-1* mutant compared to WT, which is reflected in the decreased valve cell area (Fig. 2F–H). Similar alterations were noted in fruit length in *expa15-2* and *expa15-3*. Notably, although fruit length is reduced in all three alleles due to reduced cell size, the number of valve cells is increased (Fig. 2, Figs. S2, S3, S4). However, this increased number of cells does not compensate for overall fruit length. In addition, in the *expa15-2* and *expa15-3* alleles, a reduction in seed number is observed (Figs. S2, S3). In summary, the results indicate that *EXPA15* contributes to cell expansion in fruit valves.

### The lack of *EXPA15* can affect carpel fusion

**Fig. 3:**
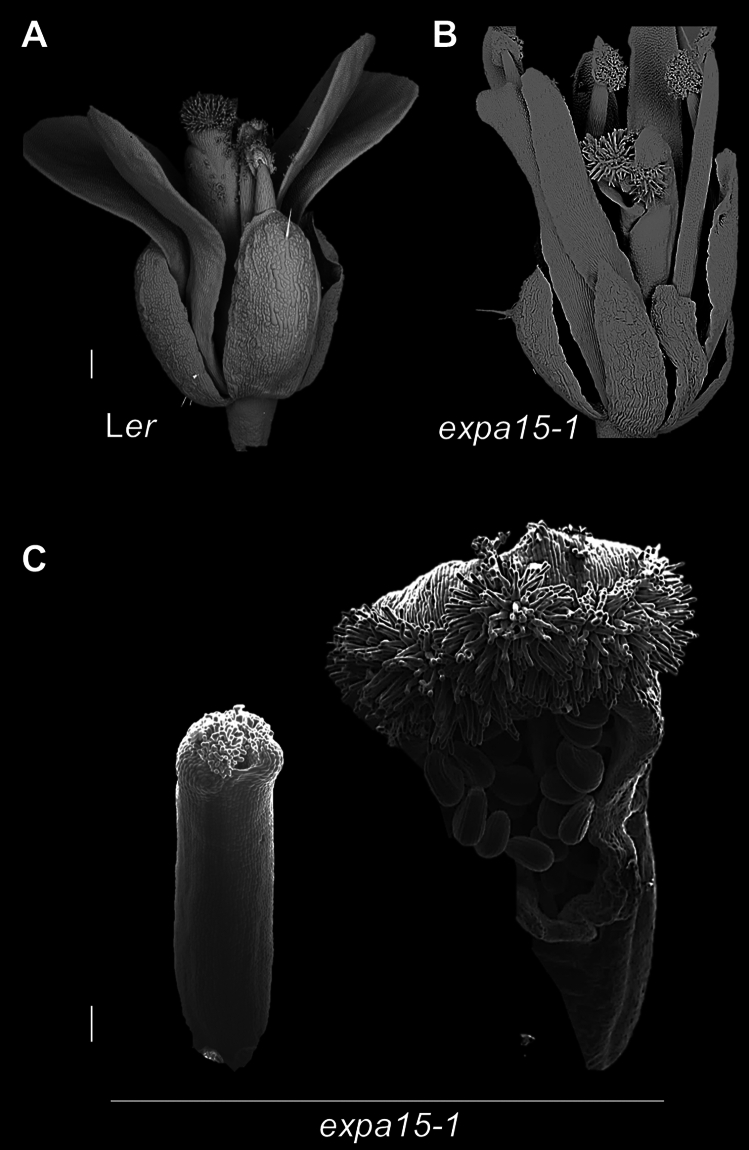
The *expa15-1* mutant is affected in carpel fusion. **A** Flower of WT. **B** Flower of *expa15-1* with unfused carpels. **C** Gynoecium of the *expa15-1* mutant with unfused carpels in the apical part, and exposed ovules can be seen. Scale bars: 200 µm in **A**–**B**; 100 µm in **C**

It is worth mentioning that the patterning of most gynoecia in the *expa15-1* mutant is normal compared to WT, though in a small frequency (around 5%), gynoecia with three valves have been observed (Fig. S5). In addition, we observed that some of the gynoecia of the *expa15-1* mutant showed affected carpel fusion (very low frequency; in around the first 8–10 flowers in the complete plant) (Fig. 3), which is a similar phenotype that has been reported for mutations in the *SPATULA* (*SPT*) gene (Alvarez and Smyth 1999; Heisler et al. 2001). This might suggest that there is a genetic interaction between *SPT* and *EXPA15*.

### *SPATULA* and *EXPA15* have a genetic interaction to promote carpel fusion

**Fig. 4:**
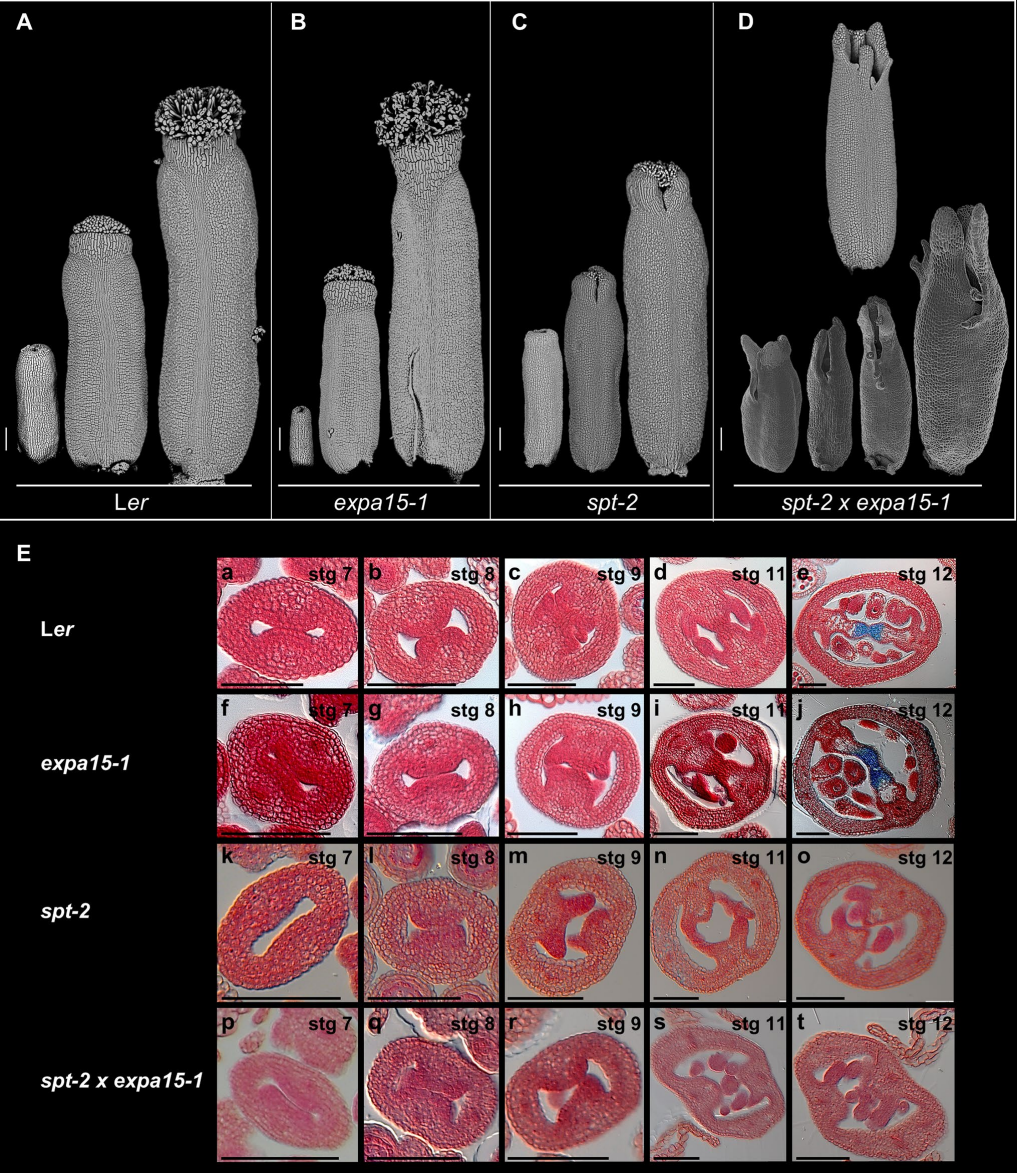
The *spt expa15* double mutant shows developmental defects in gynoecium development. **A**–**D** Scanning microscopy images of gynoecium development in WT, *expa15-1*, *spt-2* and *spt-2 expa15-1* double mutant. **E** Transverse gynoecia sections at different stages in WT, *expa15-1*, *spt-2* and *spt-2 expa15-1* mutant (**a**–**t**). Scale bars 100 µm in **A**–**D**; 10 µm in **E** (**a**–**t**)

To investigate whether there is a genetic interaction between *SPT* and *EXPA15* in gynoecium formation, we generated the *spt-2* × *expa15-1* double mutant. Interestingly, we observed that some phenotypes in the *spt-2* × *expa15-1* double mutant were additive (Fig. 4). In the *spt-2* × *expa15-1* double mutant, the petal phenotype observed in the *expa15-1* single mutant can still be observed (Fig. S6). In the *spt-2* mutant, carpel fusion defects can be seen at the apical part of the gynoecium (Fig. 4C), and internally, septum fusion and transmitting tract differentiation do not occur, as has been reported (Fig. 4E) (Alvarez and Smyth 1999, 2002; Heisler et al. 2001). The unfused carpel phenotype seems to be synergistically enhanced in the double mutant, evident from the early stages of gynoecium development (Fig. 4D). Furthermore, style and stigma development defects are also synergistically enhanced, so much that the stigmatic papillae cells are hardly observed. Interestingly, a phenotype not observed in the single mutants is that in the double mutant gynoecia, sometimes the replum continues to grow and is longer than the valves (Fig. 4D, top image). In transverse gynoecia sections, no obvious defects in medial tissue development were seen in the *expa15-1* single mutant (Fig. 4E). In the *spt-2* × *expa15-1* double mutant, defects in medial tissue development and fusion are as in the *spt-2* single mutant.

**Fig. 5:**
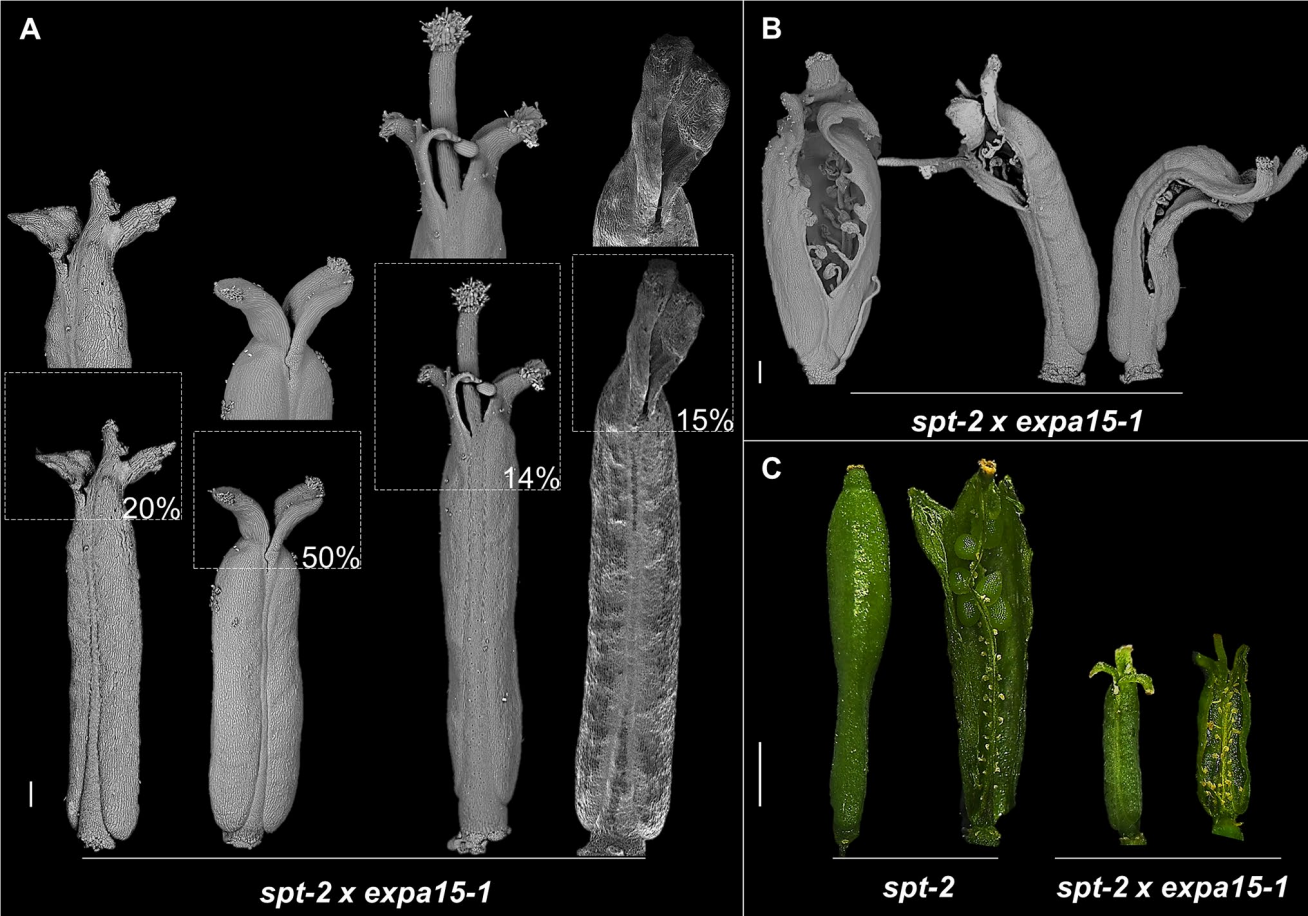
The *spt-2* × *expa15-1* double mutant produces a sterile fruit and an incorrect fusion in the apical part of the gynoecium. **A** Different fruits of the *spt-2* × *expa15-1* double mutant with morphological phenotypes such as a split style, elongated replum formation and a reduced or lack of stigma formation. **B** Most severe phenotypes seen in the *spt x expa15-1* double mutant; carpels largely unfused. **C** View of a fruit showing defects in medial tissue formation and seed-set in the *spt-2* single and *spt-2* × *expa15-1* double mutant. Scale bars: 200 µm in **A**, **B**; 1 mm in **C**

During fruit development, the fruits of the *spt-2* × *expa15-1* double mutant do not develop seeds, in contrast to seed development in the single mutants (Fig. 5). Furthermore, the apical fusion defects seen in the gynoecium can still be seen in the fruit (Fig. 5). Different fruit morphologies were observed such as the development of an elongated split style in different degrees of severity, unfused carpels with a replum that continues to grow, as well as a large medial cleft, probably as a consequence of the synergistic effect of the single mutants.

In summary, the results suggest a genetic interaction between *SPT*-*EXPA15* is required for carpel fusion and coordination of apical gynoecium and fruit development. On the other hand, the results suggest that *EXPA15* through an independent pathway contributes to petal cell expansion and valve cell expansion in the fruit.

### *SPT *indirectly can affect *EXPA15* expression during gynoecium development

**Fig. 6:**
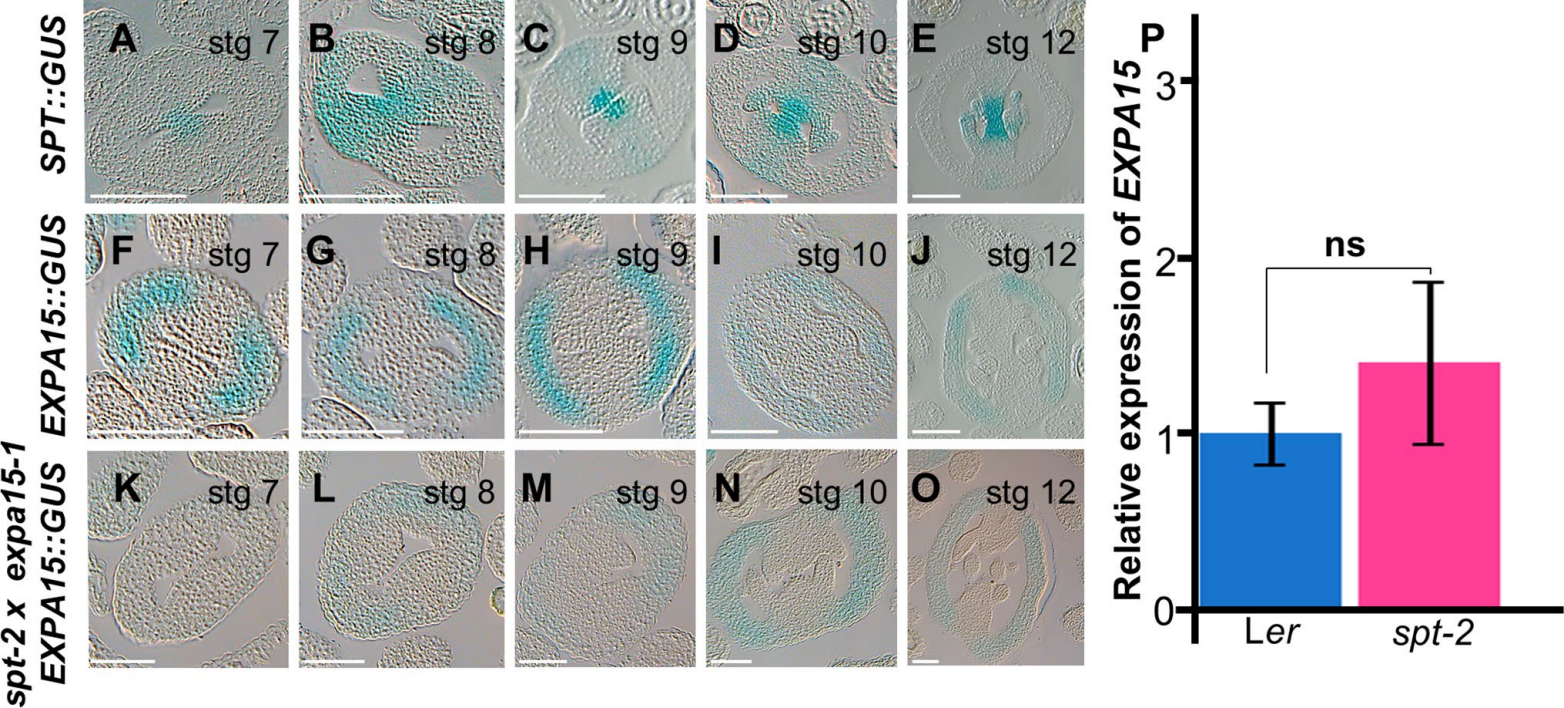
SPT could regulate *EXPA15* in early stages of gynoecium development. **A**–**E** *SPT::GUS*, **F**–**J** *EXPA15::GUS* and **K**–**O** *EXPA15::GUS spt-2* × *expa15-1* expression patterns in transverse gynoecia sections at different stages. **P** RT-qPCR expression data of the *EXPA15* gene in inflorescences of L*er* and*spt-2.* Student´s *t*-test was used to evaluate significant differences between WT and *spt-2*. Significant values are indicated as follows: *p < 0.05; ns: not significant, *p* > 0.05. Scale bar 10 µm

To further understand the genetic relationship between *SPT* and *EXPA15,* we tested whether *EXPA15* expression could be affected by the *spt* mutation. First, it has already been described that *SPT* expression in the gynoecium is localized in the medial tissue during gynoecium development (Groszmann et al. 2010) (Fig. 6A–E). We studied the CSHL_ET6476 enhancer trap line, which carries a unique insertion of an enhancer trap (ET) transposable Ds element and a GUS reporter gene (Sundaresan et al. 1995). In this case, the line is a null mutant for *expa15* (Fig. S1) and serves as a reporter line for *EXPA15*. GUS expression in the homozygous line, reflecting *EXPA15* expression, was observed very weakly at gynoecium stage 6–7 in the medial domain and strongly in the lateral domain. In later stages, strong GUS expression is observed specifically in the lateral domain of the gynoecium (Fig. 6F–J, Fig. S7). *EXPA15* expression is limited to gynoecium development, since no GUS signal is observed during fruit development, only in the fruit pedicel (Fig. S7). Interestingly, *EXPA15* expression in the medial domain at gynoecia stages 6 and 7 is almost absent in the *spt-2* × *expa15-1* double homozygous mutant background, and a disperse GUS signal in the medial tissues at stage 8. As mentioned before, at later developmental stages, strong GUS signal is only observed in the lateral domain, however, in the double mutant background, the signal is decreased. Furthermore, it appears that this decrease in *EXPA15* expression is affected from meristem formation onward, as almost no *EXPA15* expression is observed in the double mutant (Fig. S8).

Using a complementary approach to find out whether SPT regulates *EXPA15* expression, we performed RT-qPCR analyses. However, when looking at *EXPA15* expression in inflorescence tissue of the *spt-2* mutant background, no statistically significant change was observed. These data suggest, together with the lack of a clear overlap in expression patterns between *SPT* and *EXPA15*, that the effect of SPT on *EXPA15* expression in the gynoecium is indirect.

### *FRUITFUL* and *EXPA15* have a genetic interaction to promote fruit valve elongation and style morphology

**Fig. 7:**
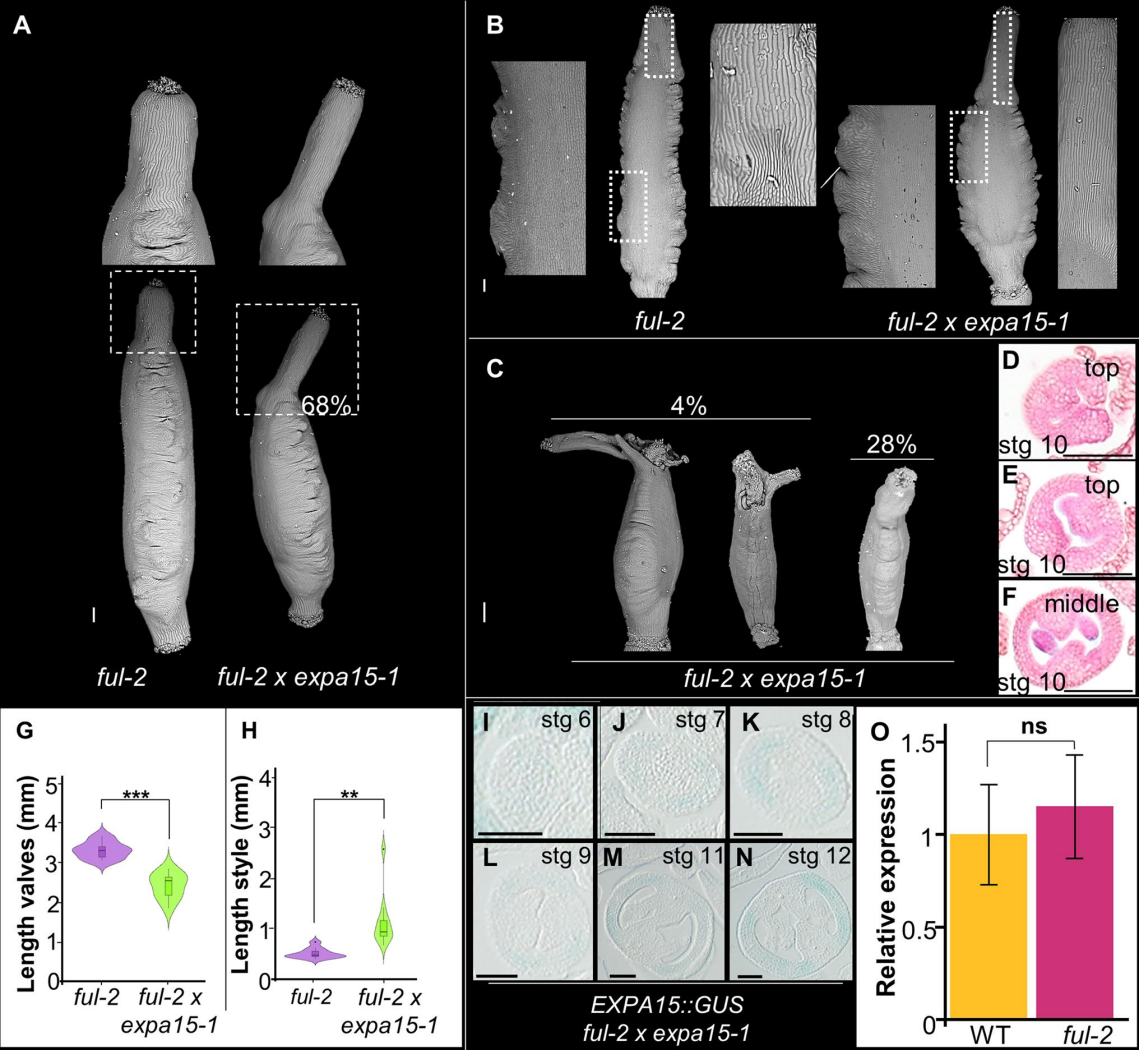
*EXPA15* and *FUL* contribute to valve and medial tissue development and *EXPA15* contributes to style development in the fruit. **A** Scanning microscopy images of *ful-2* and *ful-2* × *expa15-1* double mutant fruits. **B** Magnifications of valve and style cells in *ful*-2and *ful-2* × *expa15-1* double mutant fruits. **C** Severe phenotype in the *ful-2* × *expa15-1* double mutant fruit. **D**–**F** Transverse gynoecia sections of *ful-2* × *expa15-1* affected in medial tissue fusion. **G**, **H** Quantification of valve and style length of *ful-2* and *ful-2* × *expa15-1* double mutant. Statistical analyses were performed using a Wilcoxon test in *G* and Student´s *t* test in **H**, **O**. *n* = 10: ***, *p* < 0.001, **p < 0.01. Scale bars 200 µm in **A**–**C**; 100 µm in **D**–**F**, **I**–**N**

The above results show a genetic interaction between *SPT* and *EXPA15* in carpel fusion and apical gynoecium development. However, considering that *EXPA15* promotes cell elongation in fruit valves, as well is expressed in the valves, and *expa15-1* mutant fruits have an appearance resembling *ful-2* mutant fruits, we considered a possible relationship with the transcription factor *FUL,* which is one of the master regulators of fruit development (Gu et al. 1998). Therefore, we generated the *ful-2* × *expa15-1* double mutant, to find out if there is a genetic interaction between *FUL* and *EXPA15*. First, the *ful-2* mutant is characterized by shorter valves and a zig-zag morphology of the replum, and depending on the allele, style morphology is affected (Gu et al. 1998; Ferrandiz et al. 2000). In particular, the short valve length phenotype is exacerbated in the *ful-2* × *expa15-1* double mutant, as valve size is shorter than the single mutants (Fig. 7A, G). Interestingly, we observed that the fruit in the double mutant *ful-2* × *expa15-1* developed a longer style than the *ful-2* single mutant, something similar has been reported for other *ful* alleles (Fig. 7A) (Ferrandiz et al. 2000). It is worth mentioning that the cells of the double mutant *ful-2* × *expa15-1* are similar to those of the *ful-2* single mutant, likewise, the cells of the style are more elongated, causing the style in the double mutant to be longer (Fig. 7A, B, H). In addition, we observed in a lower frequency fruit with an unfused apex, a seemingly one-side of a split style structure and reduced stigmatic tissue (Fig. 7C). The effect of the lack of fusion of the medial tissue can be observed from the formation of the gynoecium (Fig. 7D–F). Furthermore, looking at the pattern of *EXPA15* expression in the *ful-2* × *expa15-1* double mutant, based on the GUS signal this expression is decreased in the lateral domain (Fig. 7I–N). In addition, we have performed RT-qPCR analysis to determine whether *EXPA15* expression is regulated by FUL (Fig. 7O). However, we did not observe a change in *EXPA15* expression in the *ful-2* mutant. These data confirm that there is a genetic interaction between *FUL-EXPA15* to promote fruit valve elongation and style development, but we cannot conclude that FUL regulates *EXPA15* during gynoecium development.

## Discussion

### *EXPA15* is an enzyme that contributes to cell morphology in petals

Cell expansion contributes to the shape and size of tissues and organs in floral organs and fruit (Alvarez-Buylla et al. 2010; Marshall et al. 2012; Ripoll et al. 2019; Herrera-Ubaldo and de Folter 2022). One of the enzyme groups that are involved in cell expansion or elongation are expansins (Samalova et al. 2022). In this work, we focused on the study of the expansin encoding gene *EXPA15*.

In general, in most flowering species, petals are characterized by having typical conical cells on their surface, though some variations can exist (Whitney et al. 2011). Based on our analyses, *EXPA15* contributes to the morphology of petal cells, since in the *expa15-1* null mutant (L*er* background) petal cells lost their typical conical morphology, being observed elongated and flat. However, their differentiation seems to be unaffected since the blade of the petals remain white, characteristic of the Arabidopsis petal (Irish 2008). In two additional alleles (*expa15-2 and expa15-3* in the Col-0 background), overall petal shape and size was also affected. All three alleles displayed a reduced number of cells in the petal. Notably, the alleles in the Col-0 background maintained the conical cell shape. In Petunia, it has been reported that the expansin *PhEXP1* is involved in petal size by controlling expansion in petal cells, however, no overall morphological changes were observed in cell shape when *PhEXP1* was downregulated (Zenoni et al. 2004). In conclusion, results support that in Arabidopsis, *EXPA15* functions in petal shape and size by controlling cell elongation and cell number, though apparently there is an accession related effect.

### SPATULA and EXPA15 together participate in carpel fusion in Arabidopsis

On the other hand, the Arabidopsis gynoecium is characterized by a fusion of two carpels at early stages of development. Cell proliferation and expansion is key for medial domain tissues of the gynoecium to fuse and subsequently differentiate into septum tissue, transmitting tract and subsequent formation of a style and stigma (Bowman et al. 1999; Alvarez-Buylla et al. 2010; Reyes-Olalde et al. 2013). Expansion during gynoecium development has been described to be a stage and tissue-specific phenomenon during development (Gómez-Felipe et al. 2023). Interestingly, in *expa15-1* mutants, some of the gynoecia showed incorrect carpel fusion as seen in the *spt* mutant (Alvarez and Smyth 1999, 2002; Heisler et al. 2001), and this effect is enhanced in the *spt-2* × *expa15-1* double mutant.

Based on the localization of *SPT* and *EXPA15* expression, in the gynoecium there is no or hardly any overlap, meaning that the effect of SPT on *EXPA15* is likely indirect. Only in the floral meristem, there is overlap in gene expression, though further studies would be necessary to explore if there is a direct interaction.

On the other hand, the phenotype of a split style and absence of stigma in the double mutant *spt-2* × *expa15-1* has been observed in other double mutants of SPT and its target genes *HOMEOBOX ARABIDOPSIS THALIANA 3 (HAT3*) and *ARABIDOPSIS THALIANA HOMEOBOX 4 (ATHB4)* that contribute to the apical radialization of the style (Reymond et al. 2012; Carabelli et al. 2021), as well as in mutants of transcription factors of the NGATHA family (Trigueros et al. 2009), affecting the signaling or biosynthesis of auxins and cytokinins. It is known that *EXPA15* can be regulated by type B Arabidopsis Response Regulators (type B ARRs) of the cytokinin signaling pathway, since its promoter contains DNA binding sites of this family of transcription factors (Samalova et al. 2023). In addition, SPT is known to contribute to cytokinin signaling by regulating *ARR1* and *ARR12* (Reyes-Olalde et al. 2017a, b), which could be the regulatory pathway of *SPT* on *EXPA15* and contribute to medial tissue fusion and gynoecium apical tissue development.

### A genetic interaction between *FUL* and *EXPA15* affects fruit development

The genetic interaction between *EXPA15* and *FUL* in fruit development is reflected in the additive effect of valve size reduction in the *ful-2* × *expa15-1* double mutant. However, style elongation seems to be dependent on the participation of this enzyme, variations in style elongation have also been observed independently in other *ful* mutant alleles (Ferrándiz et al. 2000). Furthermore, lack of medial tissue fusion and a split style have also been observed in the *spt-2* × *ful-2* double mutant (Groszmann et al. 2010), reflecting the role of *EXPA15* together with *SPT* and *FUL* in medial tissue formation.

Although, it is unclear by RT-qPCR whether FUL regulates *EXPA15*, it cannot be ruled out that there is a relation with FUL, as we observed a decrease of *EXPA15* signal in the background of the *ful-2* × *expa15-1* double mutant in the lateral tissue of the gynoecium.

To conclude, according to our results, the regulation of *EXPA15* appears to be complex, as it might depend on the stage and tissue in which *EXPA15*-mediated expansion is required. This complexity of expansion regulation has been described, variable outcome of the function of an EXPANSIN has been observed depending on the amount of EXPANSIN present, i.e., despite cell expansion, at high EXPANSIN concentration, cell expansion is reduced (Choi et al. 2003; Goh et al. 2014).

Nevertheless, this work contributes to the knowledge of the importance of cell expansion in floral tissues and fruit. One of the observed phenotypes is fruit size, which is an agronomic trait that can be altered without affecting the number of seeds. The modulation and specific localization of EXPANSINS could contribute to an interesting agronomic phenotype (Marowa et al. 2016; Cosgrove 2021). Future work will be necessary to better understand how these EXPANSIN-type enzymes contribute to reproductive tissue and organ development in the plant.

## Materials and methods

### Plant material and growth conditions

*Arabidopsis thaliana* plants used in this study was the *expa15-1* mutant obtained from Jan Traas (Armezzani et al. 2018; CSHL_ET6476; ABRC CS25610), *expa15-2* (ABRC GABI_556F03, *expa15-3* (ABRC CS921359), *spt-2* (Alvarez & Smyth 1999), *pSPT-6253:GUS* (Groszmann et al. 2010), *ful-2* (Ferrandiz et al., 2000), *expa15* and *spt-2* are in the L*er* background, and *ful-2, expa15-2, expa15-3* in the Col-0 background and the wild type accessions Col-0 and L*er*. The *spt-2* × *expa15-1* double mutant was generated by crossing *spt-2* with *expa15-1*, and the *ful-2* × *expa15-1* double mutant was generated by crossing *ful-2* with *expa15-1*. Double homozygous plants (*F*_3_) were obtained from phenotype segregation assays. Seeds were germinated on soil during long day conditions (16/8 h, light/dark) at 22ºC.

### Scanning electron microscopy

During reproductive development, the different lines were scanned using a Zeiss EVO40 environmental scanning electron microscope (Carl Zeiss; Oberkochen, Germany) with 25 kV beam, and the signal was collected using the SE or the BSD detector. Each plant tissue was collected and directly observed in the microscope.

### Histology and microscopy analyses

For thin tissue section analysis, inflorescences of *expa15*, *spt-2*, *spt expa15* and L*er* were collected, and the tissue was fixed in FAE solution (3.7% formaldehyde, 5% glacial acetic acid and 50% ethanol) with vacuum (15 min, 4 °C), and afterward incubated for 60 min at room temperature. The material was rinsed with 70% ethanol and incubated overnight at 4 °C in 70% ethanol, followed by dehydration in a series of ethanol dilutions (70, 85, 95 and 100% ethanol) for 60 min each. Inflorescences were embedded in Technovit 7100 (Heraeus Kulzer) according to the manufacturer's instructions. Sections (10–12 µm) were obtained on a rotary microtome (Reichert-Jung 2040, Leica, Germany). Tissue sections were stained with a solution of 0.5% Alcian Blue and counterstained with 0.5% Neutral Red as previously described (Zúñiga-Mayo et al. 2012), or with Toluidine Blue as previously described (Herrera-Ubaldo and de Folter 2018).

The *expa15-1* mutant was generated with an enhancer trap construct that contains a Ds transposon carrying a glucuronidase reporter gene (Sundaresan et al. 1995). We used the *expa15-1* line as a GUS reporter line to reflect the *EXPA15* gene expression. The inflorescences were collected and stained as previously described (Marsch-Martínez et al. 2014), with the following modifications: 30 min of vacuum and after 1.5 h in substrate at 37 ºC, we proceeded with the dehydration with ethanol. The GUS-stained inflorescences were fixed, dehydrated as described above and embedded in Technovit 7100; 10–12 µm thick sections were analyzed. Subsequently, the samples were observed using a Nomarski Leica DM4000 microscope with DIC function.

For statistical analysis, data were first tested for normality using the Shapiro–Wilk test. Then, means were compared pair-wise using either Student's t-test, Wilcoxon test or one-way ANOVA *t* test. All calculations were performed in R (R Core Team 2022).

### Quantitative real-time RT-PCR

For RT-qPCR analysis, the *spt-2*, *ful-2* and L*er* lines were collected inflorescences with floral buds only. Three biological replicates were sampled. After collection, total RNA was extracted using the Direct-zol RNA Mini Prep Plus Kit (Zymo Research, USA). Reverse transcription and amplification were performed in triplicate with a SyGreen 1-Step Go Hi-ROX qPCR kit (PCR BIOSYSTEMS, USA). RT-qPCR was performed using a real-time Open qPCR machine (Model: A1005, CHAIBIO, USA). Expression levels of target genes were normalized with *TUA2*. Data were analyzed using the 2^−ΔΔCT^method (Livak and Schmittgen 2001). The following primers were used: *EXPA15* (AT2G03090) F:5’- CTTCTGTAGGAAACAGGGACAAC-3’ and R: 5’- CCTCCGCTTCATCATTTCGATC-3’, and *TUA2* (AT1G50010) F: 5’-GGTTCCAGGTTTGTCACTCGTT-3’ and R: 5’-CCGAGAAGGTAAGCATCATGCG-3’.

### Supplementary Information

Below is the link to the electronic supplementary material.

Supplementary file 1 (PDF 1128 kb)
