# Supplementary material for: EXPANSIN15 is involved in flower and fruit development in Arabidopsis
Source: Plant Reprod. 2024 Jan 29;37(2):259–70. doi: 10.1007/s00497-023-00493-4 (PMC11180156; doi:10.1007/s00497-023-00493-4)
Supplement: Supplementary file 1 — (PDF 1128 kb) [file 497_2023_493_MOESM1_ESM.pdf]

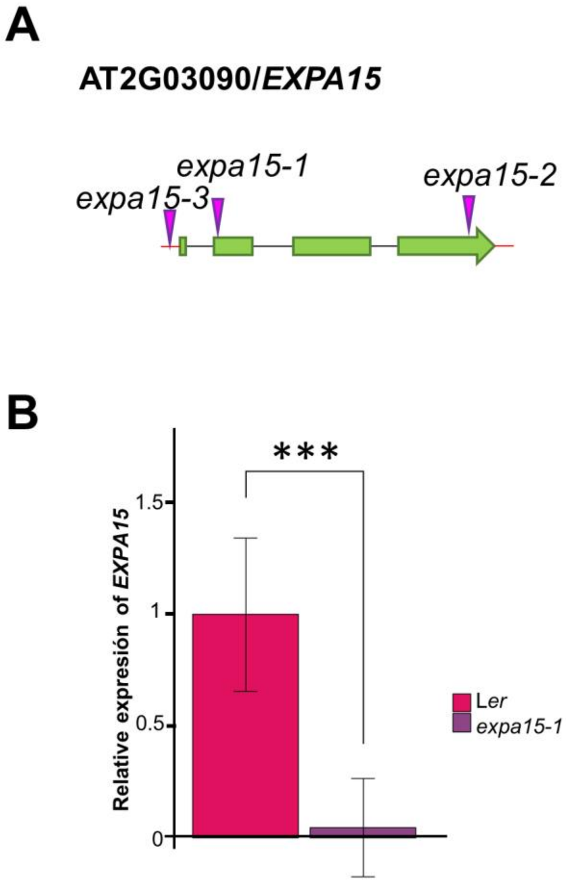

Fig. S1. Gene diagram of *EXPA15* with the mutant alleles studied. A) schematic of the genomic region of *EXPA15* gene. Exons are indicated by green boxes, the 5′- and 3′- with red lines and introns by thick black lines. The triangles indicate the position of the insertions of the *EXPA15* mutant alleles studied. B) RT-qPCR expression data of the *EXPA15* gene in inflorescences of L*er* and *expa15-1,* showing that the *expa15-1* allele is a null mutant. Student´s t-test was used to evaluate significant differences between L*er* and *expa15-1*. Significant values are indicated as follows: ***p<0.001; ns: not significant, P > 0.05.

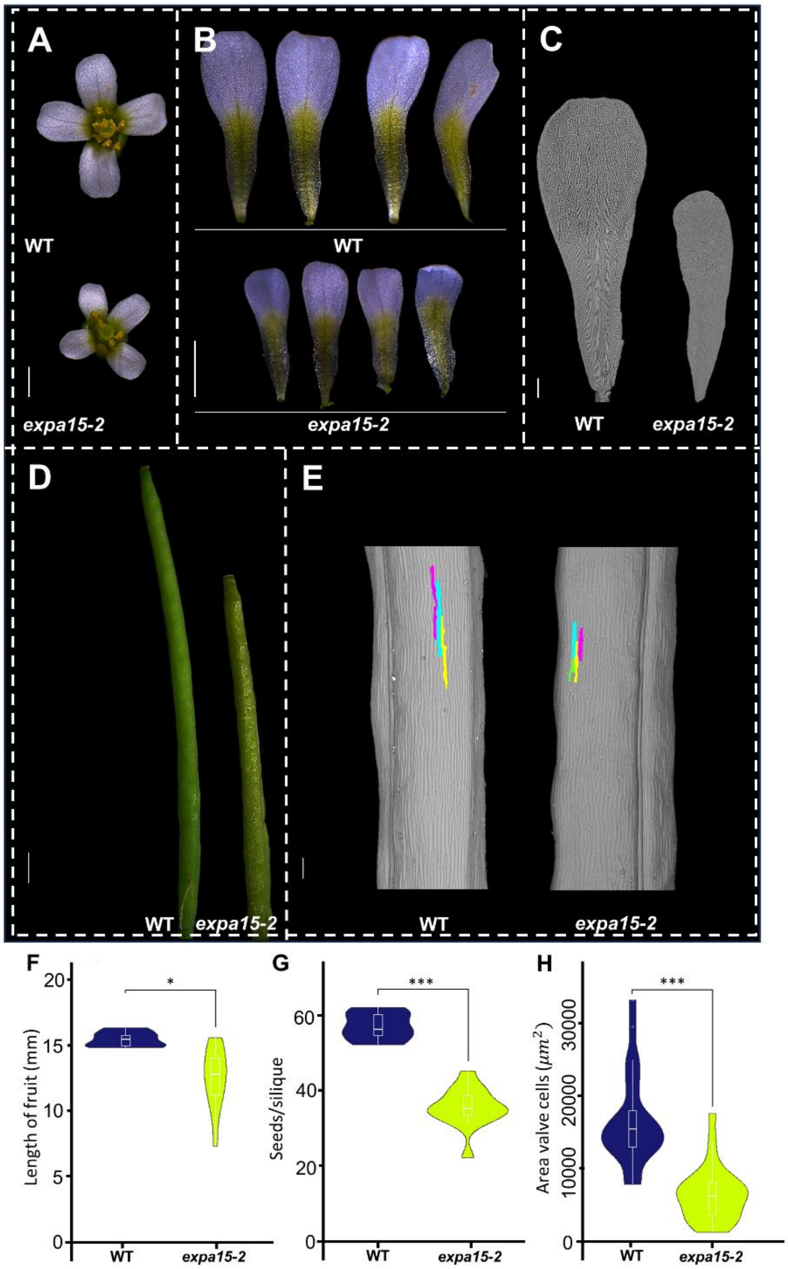

Fig. S2. Phenotypes in the *expa15-2* allele in the Col-0 background. A) Flower of wild-type Col-0 and *expa15-2*. B) Smaller petals in *expa15-2* compared to Col-0. C) The petal cells of *expa15* are similar to WT in the adaxial part of petals. D) Images of fruits of Col-0 and *expa15-2*. E) Scanning microscopy images of Col-0 and *expa15-2* fruits. F-H) Analysis on fruits of Col-0 and *expa15-2*. F) Fruit length. G) Number of seeds per silique. H) Analysis of fruit valve cell area in Col-0 and *expa15-2*. Note: Images/measurements of WT samples are the same as in Fig. S3. Statistical analyses were performed using a Wilcoxon test. n=15 in F,H, n=5 in H: *p<0.05, ***p<0.0001, ns: not significant, p > 0.05. Scale bars:1 mm in A-B, D; 200 µm in C; 100 µm in E.

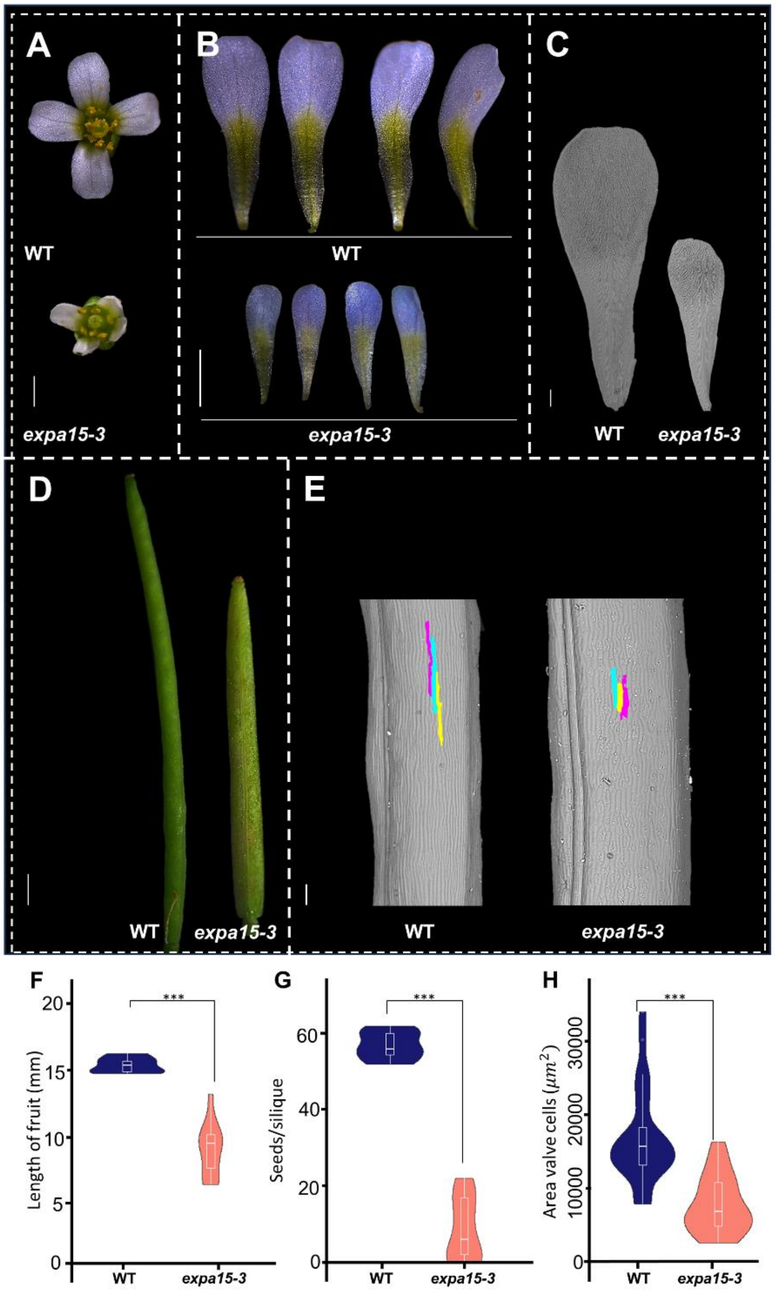

Fig. S3. Phenotypes in the *expa15-3* allele in the Col-0 background. A) Flower of wild-type Col-0 and *expa15-3*. B) smaller petals in *expa15-3* compared to Col-0. C) The petal cells of *expa15-3* are similar to WT in adaxial part of petals. D) Images of fruits of Col-0 and *expa15-3*. E) Scanning microscopy images of Col-0 and *expa15-3* fruits. F-H) Analysis on fruits of Col-0 and *expa15-3*. F) Fruit length. G) Number of seeds per silique. H) Analysis of fruit valve cell area in Col-0 and *expa15-2*. Note: Images/measurements of WT samples are the same as in Fig. S2. Statistical analyses were performed using a Wilcoxon test. n=15 in F,H, n=5 in H: ***p<0.0001, ns: not significant, p > 0.05. Scale bars:1 mm in A-B, D; 200 µm in C; 100 µm in E.

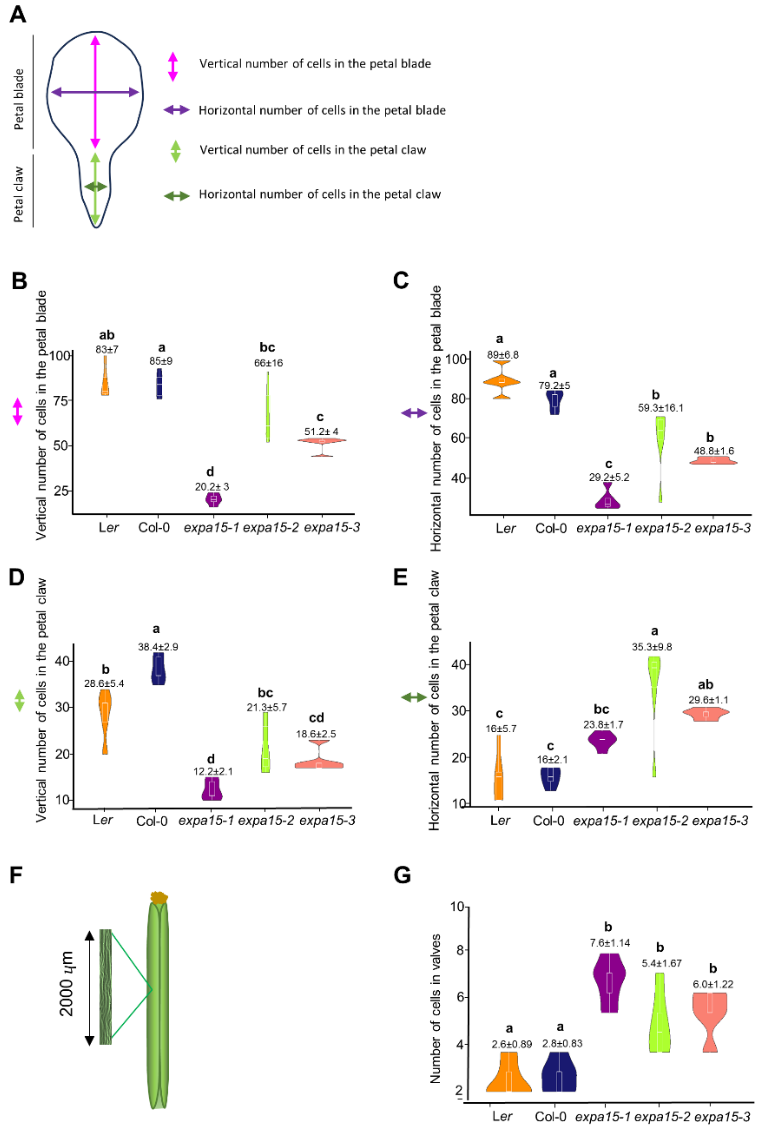

Fig. S4. The cell number in petal and fruit in the mutant alleles *expa15-1*, *expa15-2* and *expa15-3* is affected compared to the WT. A) Annotation scheme of areas of quantification of cells in the petal. B) Quantification of vertical lineal number of cells in the petal blade in L*er*, Col-0, *expa15-1*, *expa15-2* and *expa15-3*. C) Quantification of horizontal lineal number of cells in the petal blade in L*er*, Col-0, *expa15-1*, *expa15-2* and *expa15-3*. D) Quantification of vertical lineal number of cells in the petal claw in L*er*, Col-0, *expa15-1*, *expa15-2* and *expa15-3*. E) Quantification of horizontal lineal number of cells in the petal claw in L*er*, Col-0, *expa15-1*, *expa15-2* and *expa15-3*. F) Schematic of Arabidopsis fruit showing the measurement of quantified valve cells in a vertical line. G) Quantification of valve cells in a vertical line of 2000 µm length in L*er*, Col-0, *expa15-1*, *expa15-2* and *expa15-3*. Statistical differences detected by one-way ANOVA and Tukey HSD on stage 13-14 flowers at $p < 0.05$ are represented by different lowercase letters on box plots. n = 5.

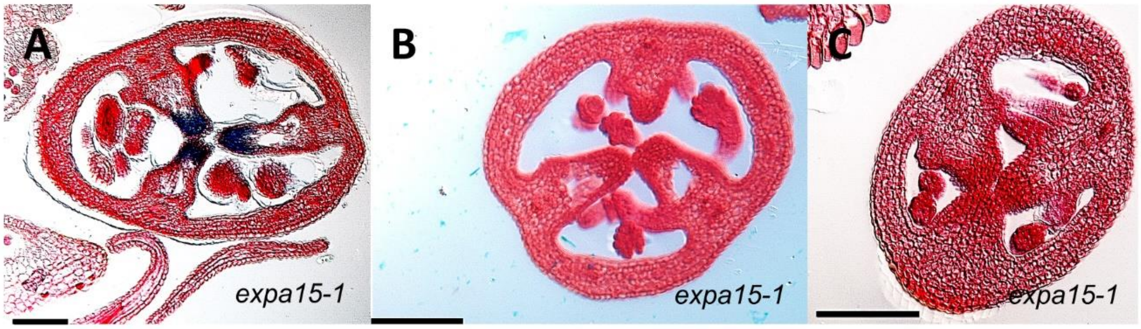

Fig. S5. The *expa15-1* mutant occasionally shows a gynoecium with three carpels. A-C) Transverse sections of Arabidopsis gynoecia stained with Neutral red and Alcian blue of the *expa15-1* mutant showing three carpels at low frequency (5%; 2 flowers per inflorescence of around 40 flowers). Scale bars 10 µm.

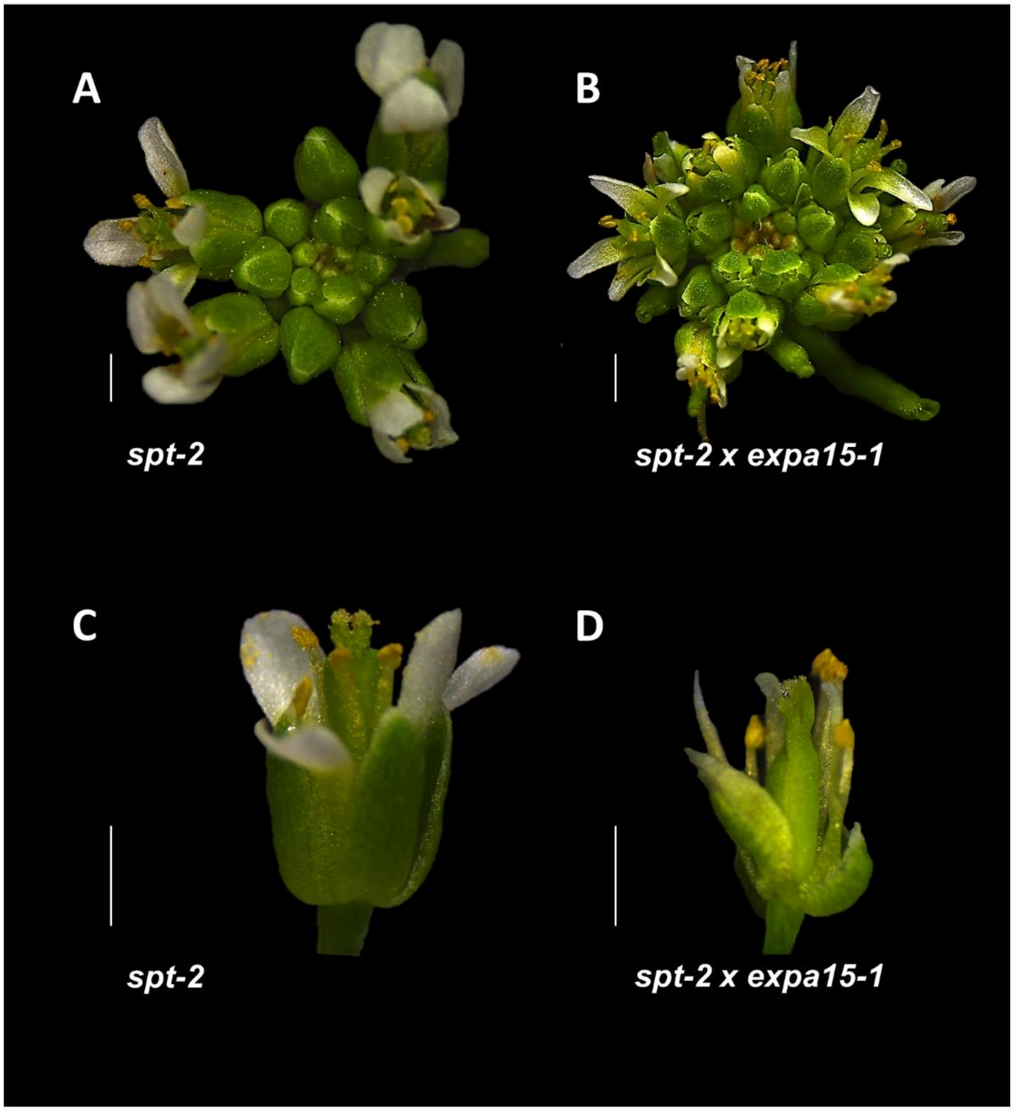

Fig. S6. The *spt-2 x expa15-1* double mutant presents a stronger phenotype than the single mutants. A, B) View of *spt-2* and *spt-2 x expa15-1* double mutant inflorescences. C, D) View of *spt-2* and *spt-2 x expa15-1* double mutant flowers. Scale bars 1 mm.

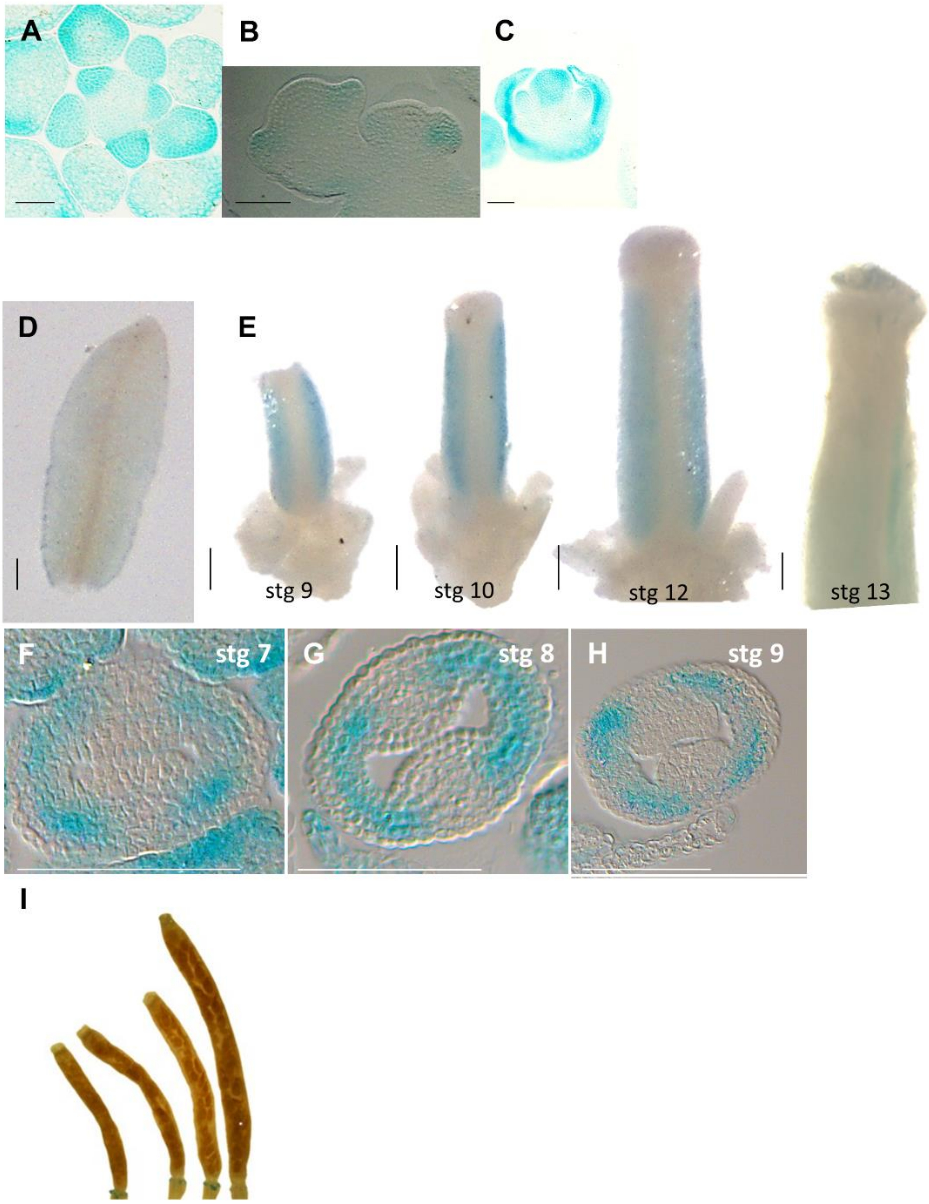

Fig. S7. Expression pattern of *EXPA15* in the shoot apical meristem, floral meristem, petals, gynoecium, and fruit. A, B) Expression pattern of *EXPA15* in the shoot apical meristem in a vertical and horizontal view after overnight GUS reaction. C) Expression pattern of *EXPA15* in the floral meristem. D) Expression pattern of *EXPA15* in the petal. E) Expression pattern of *EXPA15* in gynoecia until stage 13. F-H) Expression pattern of *EXPA15* in gynoecia cross sections. I) Absence of *EXPA15* signal in fruits. Scale bars: 50 µm in A-C; 1 mm in D-E; 10 µm in F-H.

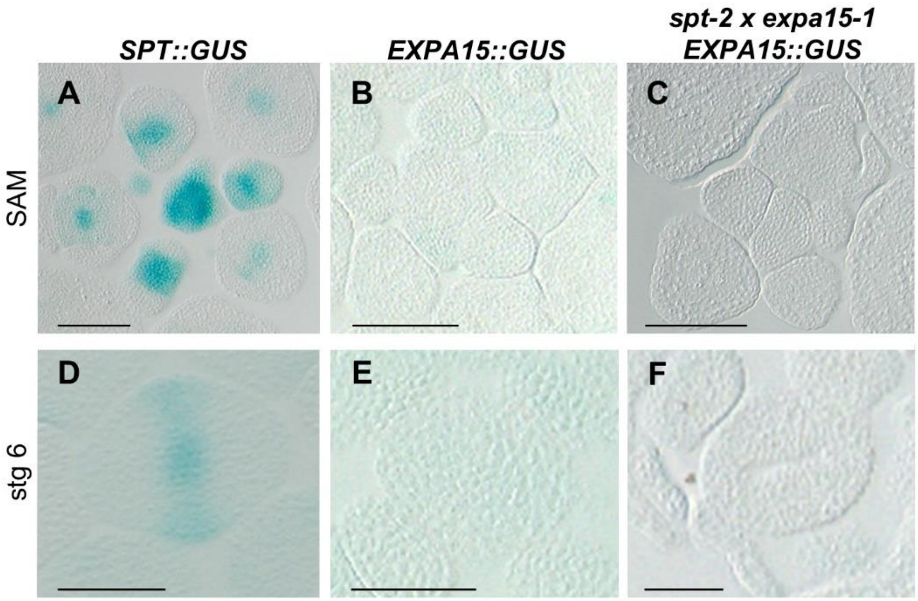

Fig. S8. *EXPA15* expression appears to be affected during meristem development in the *spt expa15* double mutant. A-C) Expression pattern of *SPT* and *EXPA15* in WT, and *EXPA15* expression in the *spt-2 expa15-1* double mutant in transverse sections of the meristem after two hours of GUS reaction. D-F) Expression pattern of *SPT* and *EXPA15* in WT, and *EXPA15* in the *spt-2 expa15-1* double mutant in transverse gynoecia sections at stage 6. Scale bars: 100 µm in A-C; 50 µm in D-F.

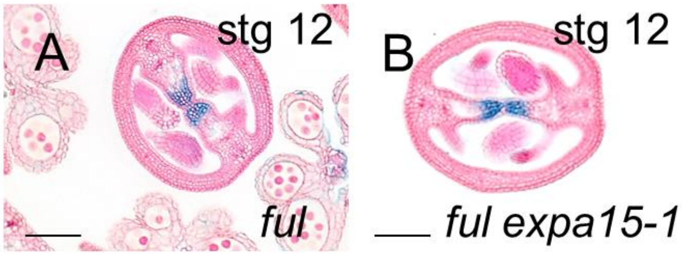

Fig. S9. Medial tissues develop normally in most gynoecia of the *ful expa15* mutant. A-B) Transverse gynoecia sections stained with Neutral red and Alcian blue at stage 12 of *ful-2* and the *ful-2 x expa15-1* double mutant. Scale bars 100 µm.
